# The Effects of Salt Stress on Germination, Seedling Growth and Biochemical Responses of Tunisian Squash (*Cucurbita maxima* Duchesne) Germplasm

**DOI:** 10.3390/plants11060800

**Published:** 2022-03-17

**Authors:** Neji Tarchoun, Wassim Saadaoui, Najla Mezghani, Ourania I. Pavli, Hanen Falleh, Spyridon A. Petropoulos

**Affiliations:** 1 High Agronomic Institute of Chott Mariem, University of Sousse, Sousse 4042, Tunisia; wessaadaoui@gmail.com (W.S.); najla_mezghani@yahoo.fr (N.M.); 2 National Gene Bank, Boulevard du Leader Yasser Arafat, ZI Charguia 1, Tunis 1080, Tunisia; 3 Department of Agriculture, Crop Production and Rural Environment, University of Thessaly, Fytokou Street, 38446 Volos, Greece; ouraniapavli@uth.gr; 4 Centre of Biotechnology of Borj Cedria, BP 901, Hammam-lif 2050, Tunisia; hanenfalleh@gmail.com

**Keywords:** salinity stress, proline, seed germination, MDA, *Cucurbita* sp., landrace

## Abstract

Salt stress is considered as one of the most common abiotic stresses reducing the productivity and fruit quality of crop plants. The present study was carried out to assess the salt tolerance among 15 local squash (*Cucurbita maxima* Duchesne) landraces. Different salt (NaCl) concentrations of 0, 100, 200 and 300 mM were selected in order to evaluate the response of the study germplasm to salt stress based on 12 agronomic parameters and 3 biochemical traits, proline, malondialdehyde (MDA) and chlorophylls. A varied effect of the salt stress level was observed among the studied landraces based on germination potential, as well as on growth and biochemical parameters at seedling stage. Results showed that all landraces were drastically affected at high stress level with a significant variation in their stress response, indicating the existence of considerable genetic variability. Landraces “746” and “747” were the best performing cultivars across stress levels, whereas “1007”, “1008” and “1009” were the most negatively affected. Based on the tested landrace performance, four landraceswere selected and further evaluated at biochemical level, focusing on the determination of compounds that play a key role in the ability to withstand salt stress. The mean MDA content across landraces was generally increased in stressed plants, as compared to the control treatment; the increase was attributed to a peak in MDA content at specific stress levels. In particular, “746” and “1007” showed the maximum content at 100 mM NaCl, while in landrace “751”, MDA content reached its peak at 300 mM NaCl. In addition, the response of most landraces to salt stress involved an increase in free proline content, with the exception of “746”, with the maximum content being observed either at 200 mM (“748” and “751” landraces) or at 300 mM NaCl, where only “747” expressed the highest content. These findings can be extrapolated into efforts to develop more salt-tolerant squash landraces and exhaust the possibilities of using saline water or soils under changing climate conditions.

## 1. Introduction

The genus *Cucurbita*, belonging to the family Cucurbitaceae, includes several economically and nutritionally important vegetable crops cultivated worldwide [1]. It contains five domesticated species, namely *C. argyrosperma* Huber, *C. ficifolia* Bouché, *C. maxima* Duch., *C. moschata* Duch. and *C. pepo* L. [2]. *Cucurbita maxima* Duch. is an allogamous and extremely diverse species that originated in South America from the wild progenitor *C. maxima* ssp. *andreana* (Naud.). The species contains a series of squash ecotypes, whose dispersal is attributed to Spain, which acted as a bridge between South America and Europe, and from there dispersed into other continents [3,4], while its domestication has been regulated by hybridization, introgression and gene flow processes [4]. 

In Tunisia, squash (*Cucurbita maxima* Duch.) is usually cultivated in small farms. At a local level, the germplasm employed for cultivation refers to landraces produced by open pollination or farmer mass selection and maintained by local farmers. Tunisia is considered to be one of the most important diversity centers of cultivated cucurbits [5], with squash landraces being produced in various cultivation zones (North East, Central West, Cap-Bon, Sahel, Siliana, Monastir, Tunisia). Given the importance of local squash production, in recent years, several efforts have addressed the issues of collection, ex-situ characterization and maintenance of the local germplasm accessions [6]. In this concept, the studies of ISA CM (High Agronomic Institute of Chott Mariem) revealed important parameters related to the agronomic performance and valorization of local landraces [7], while the National Gene Bank of Tunisia is currently conducting a breeding program focusing on collecting squash landraces and wild relatives from selected areas that represent the cultivating zones of Tunisia. Recent studies have revealed that the local germplasm of *C. maxima* is highly adaptive to diverse agro-climatic conditions and possesses considerable genetic variability for important agronomic traits as well as traits related to fruit size and fruit quality [6,7]. 

Many authors reported that salinity is considered as one of the most common abiotic stresses reducing the productivity and fruit quality of crop plants [8,9,10]. The major contributing factors to soil salinization include climate change, leading to land degradation and desertification [11], as well as the poor quality of irrigation water and irrational fertilization management, which results in reduced productivity in either irrigated or rainfed farming systems [12]. The compromised crop performance is the result of a combined osmotic and ionic stress that induces complex interactions at morphological, physiological, biochemical and molecular level [13,14], thus leading to altered photosynthetic activity, detoxification capacity, energy state and plant cellular homeostasis [15,16,17]. In particular, salt stress is interlinked with lipid peroxidation in cellular membranes, DNA damage, protein denaturation, carbohydrate oxidation, pigment breakdown and impairment of enzymatic activity, as well as metabolic adaptations, mainly involving the accumulation of osmolytes [16]. Osmolyte accumulation acts in favor of cell water uptake and cell turgor maintenance, stabilization of membranes, enzymes and proteins and the reduction of oxidative damage due to decreased Reactive Oxygen Species (ROS) levels, thereby contributing to redox balance [18]. Well known examples of metabolites with an osmoprotective function under salt stress conditions are certain amino acids, mainly referring to proline, and glycine betaine, belonging to the group of quaternary amines [19]. 

In salt-sensitive species, the stress effects vary considerably depending on the extent and duration of the stress but also on factors relating to plant characteristics [20,21,22,23,24]. Although most plant species are salt-sensitive at all stages of their lifecycle, their sensitivity differs among growth stages [21], with seed germination being viewed as the most critical stage when salt stress impairs water absorption during seed imbibition and turgescence [25]. At this stage, salt stress is expressed through the reduction in germination percentage, delayed germination rate and inhibited tissue elongation [23,26]. The increased Na+ and Cl- ion concentration induces ionic toxicity, oxidative stress and nutritional imbalance as well as water stress by lowering the osmotic potential of soil solution, ultimately leading to the inhibition of germination in many species [8,27,28]. Salt stress further affects the ultrastructure of root cells and severely inhibits root growth during early seedling growth [29], as compared to shoot growth, in various plant species, including tomato [30], chickpea [31], lentil [32] and lettuce [33]. However, such salt stress responses are highly subjected to both species and cultivar dependency, with great variability being reported [34,35,36].

In Tunisia, soil salinization is gradually increasing due to the scarcity of rains and the increase in evapotranspiration, adversely affecting plant germination, growth, development and fruit setting in salt-affected soils, as is the case of semiarid regions [37]. In squash, salinity is a factor that severely limits crop growth and productivity while at the same time deteriorating fruit quality. Despite the acquired knowledge in relation to the salt stress response in a plethora of plant species [33,38,39], there is a gap in relevant research fields for squash. Considering that the development of salt-tolerant germplasm is one of the most effective means for enhancing squash production in saline soils, this study aimed at investigating the response of squash germplasm to salt stress at the germination stage and examining the potential of selecting salt-tolerant genotypes at the laboratory level early. As such, 15 Tunisian squash landraces were subjected to NaCl-induced salinity stress at varying stress levels (0, 100, 200 and 300 mM of NaCl), and their response was assessed on the basis of traits related to seed germination and seedling growth potential. Moreover, based on the results related to salt stress response, four landraces were selected and further assessed in terms of biochemical parameters that are routinely employed as indicators of salt tolerance, namely malondialdehyde (MDA), free proline and chlorophyll a and chlorophyll b content.

## 2. Results

### 2.1. Effect of Salt Stress Level on Traits Related to Seed Germination and Seedling Growth Potential 

In order to evaluate the response of 15 Tunisian squash germplasm to salt stress (see the corresponding Table in Section 4), seeds were germinated on solutions differing in NaCl concentration (0, 100, 200 and 300 mΜ), and seed and seedling parameters were evaluated based on 12 criteria (see the corresponding Table in Section 4). The obtained data revealed a significant effect of the salt stress level (*p* < 0.001) on the germination and seedling growth potential of the tested squash landraces, with the stress effects being in most cases analogous to the stress level applied, therefore leading to most drastic effects at high stress levels for all the traits under study (Table 1 and Table 2).

The final germination percentage, evaluated on the seventh day after salt stress initiation, was strongly affected by the stress level, thus showing decreasing trends as NaCl increased (GP: 86.35%, 23.33%, 15.00% and 8.40% at 0 mM, 100 mM, 200 mM and 300 mM NaCl, respectively) (Table 2). The analysis of data further revealed significant differences in shoot (SL) and root length (RL) among the stress levels applied, with most profound effects being noted at the high stress level (Table 2). Accordingly, the shoot/root ratio (SRR) was considerably affected by the stress level, presenting a gradual increase as NaCl concentration increased (ranging from 1.28 to 3.58), which reflects the fact that root length was more profoundly affected than shoot length under stress conditions (Table 2). Such drastic effects were also noted in shoot (SFW) and root fresh weight (RFW), which were significantly affected by the stress level. In particular, both SFW and RFW showed decreasing trends as NaCl concentration increased, with the respective values ranging between 0.90–0.31 g and 0.14–0.04 g for shoots and roots (Table 2). 

In addition, a significant effect of the salt stress level on the germination stress tolerance index (GSTI) was also recorded, with the effects being proportional to the stress level applied. In particular, GSTI decreased as NaCl concentration increased (ranging from 0.29% to 0.09%), thus reflecting the differential effects of the various stress levels on seed germination potential (Table 2). Accordingly, the shoot length tolerance index and root length stress tolerance index (SLSTI and RLSTI) declined with increasing stress level. The effects of stress on root length were profoundly evidenced at higher stress levels where significant differences between the levels of 200 mM and 300 mM of NaCl were recorded, whereas in the case of shoot length, no significant differences in SLSTI values were recorded between the indicated stress levels (e.g., 200 mM and 300 mM of NaCl). In agreement with the abovementioned data for shoot and root fresh weight, the most drastic effects were noted at RLSTI compared to SLSTI, as a result of the most severe effects of salt stress on seedling root length, which are expressed with lower values of the RLSTI index (Table 2).

### 2.2. Effect of Landrace on Traits Related to Seed Germination and Seedling Growth Potential 

The data underline the significant effect of landrace (*p* < 0.001) on the germination and seedling growth potential of squash under salinity stress conditions (Table 1 and Table 3). The differential response of the landraces to salt stress was evidenced for all the traits under study, while the analysis showed RFW, SRR, SLR, RRL and GSTI as the most variable traits (CV > 20%) (Table 1). 

Germination was considerably affected by the landrace, as evidenced by the mean GP of landraces under study (Table 3), which ranged from 49.33% to 16.75% in landraces “747” and “1008”, respectively. Although such values suggest a high salt tolerance ability and sensitivity for “747” and “1008” landraces, respectively, they are also attributed to their innate germination potential, as evidenced by the differences in GP in the respective control treatments (97.33% and 67.00% for “747” and “1008” respectively) (Table 4). Moreover, the landraces “746”, “752”, “745”, “748”, “749” and “750” were characterized by a mean GP of 39.50%, while the other landraces presented intermediate GP values ranging from 31.25% to 21.50% (Table 3). In relation to seedling growth potential, the effects of stress were consistently more severe on roots than on shoots (Table 3). Although the stress effects were obvious in all landraces, their response to salinity differed substantially. As such, “1006” presented the highest values for both SL and RL, while the lowest respective values were noted in “1007” and “1008”. Regarding the SRR, “753” and “1007” presented the highest and lowest values, respectively. Accordingly, significant differences were also observed for SFW and RFW, with “752” showing the highest value for both traits, while the lowest values were recorded in “1008” and “746” landrace, respectively (Table 3). 

In agreement with the abovementioned findings, a considerable variation for the GSTI, SLSTI and RLSTI was also recorded (Table 3). Based on GSTI, “747” followed by “746” were proved to be the best performing landraces. In relation to SLSTI, “747” followed by “1006”, “753” and “746” showed the highest tolerance to salt stress, whereas “1006” ranked as the best performing landrace in terms of RLSTI. In contrast, “1007”, “758” and “759” were proved to be the most salt-sensitive based on GSTI, SLSTI and RLSTI values (Table 3).

### 2.3. Effect of the Landrace and the Salt Stress Level on Traits Related to Seed Germination and Seedling Growth Potential 

Based on the analysis of variance applied on individual data, all traits related to germination and seedling growth potential under salt stress conditions were differentially affected by the landrace and the salt stress level applied (Table 4). In the absence of stress, germination was considerably affected by the landrace, thus substantiating a variable germination potential per se, which could be mainly attributed to the median longevity of seeds whose fruits were harvested at different periods (2018 to 2020). Among landraces, “746” and “1005” presented the highest and lowest GP under normal conditions (98.66 % and 63.00 %, respectively) which also justifies that seed longevity is a genotype-dependent variable (Table 4). Upon stress, the germination of all landraces was severely affected, with the effects of stress being in general analogous to its level. At 100 mM NaCl, “745” proved to be the most capable landrace of retaining a high germination rate (50.00%), while “746”, “747” and “749” also showed relatively high GP values (≥40%). In contrast, “1007”, “1008” and “1009” landraces, which were of medium-high innate germinability (67.00–96.00%), were incapable of germination at all stress levels (Table 4). Moreover, at 200 mM NaCl, “746” and “747” presented the highest GP (35.00 %), whereas “754” and “1006” ranked as the landraces with the lowest GP values. Finally, at 300 mM NaCl, “747” followed by “746” proved to be the best performing landraces (25% and 20%, respectively). Interestingly, “745”, although showing superior performance at 100 mM NaCl, suffered great losses at both 200 mM and 300 mM NaCl (15.00% and 5.00%, respectively). Furthermore, the response of most landraces to salt stress involved a drastic reduction in both SL and RL, with the latter parameter being more pronouncedly affected in the majority of the tested landraces (Table 4). At all stress levels, “1006” presented the highest values for SL and RL, thus proving its ability to withstand even high levels of salt stress. In contrast, “750” consistently showed low values for SL and RL—a finding that could be partly attributed to its low growth potential per se, as evidenced by the respective values in the control treatments (6.83 and 5.10 mm, respectively) (Table 4). In agreement with these findings, the SFW and RFW followed a decreasing trend as NaCl concentration increased. The highest values for these traits were recorded in “752” both in control and stress conditions, whereas the lowest values were noted in “1006” and “746” for SFW and in “747” for RFW. Although affected at all stress levels, the SRR was more profoundly affected at 300 mM NaCl, with the landraces showing varying values ranging from 10.19 to 1.27. The highest SRR was noted in “753”, thus reflecting the more drastic effect of salt stress to the roots than in shoots (Table 4). 

**Figure 1:**
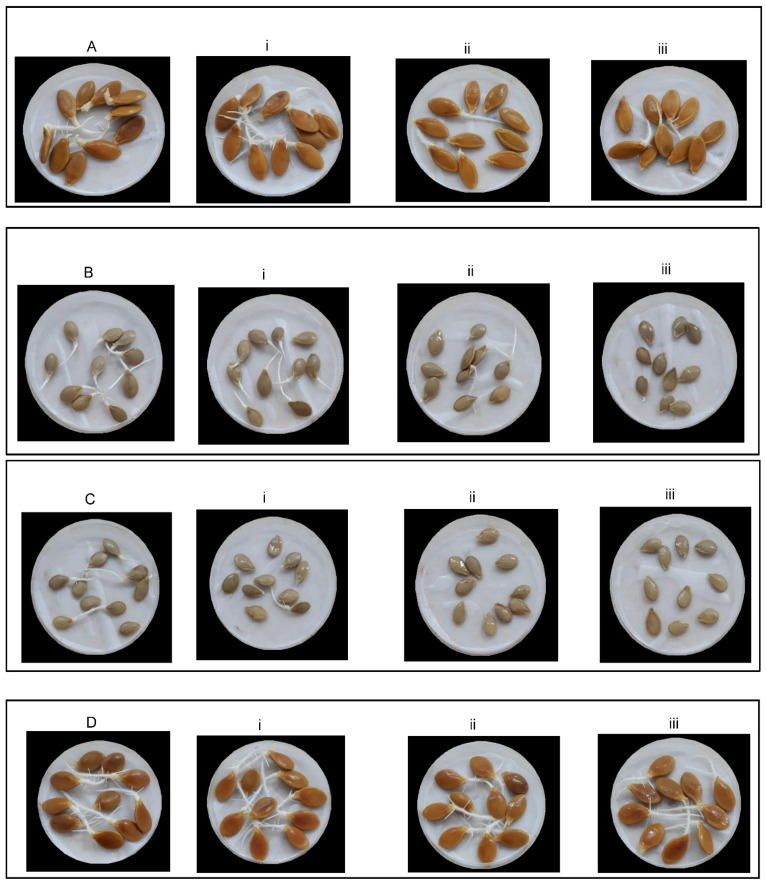
Effect of salt stress treatment on four selected landraces representing the main types of cultivated squash: (**A**): “748” control 0 mM NaCl, (**i**) 100 mM NaCl, (**ii**) 200 mM NaCl, (**iii**) 300 mM NaCl; (**B**): “747” control 0 mM NaCl, (**i**) 100 mM NaCl, (**ii**) 200 mM NaCl, (**iii**) 300 mM NaCl; (**C**): “751” control 0 mM NaCl, (**i**) 100 mM NaCl, (**ii**) 200 mM NaCl), (**iii**) 300 mM NaCl; (**D**): “746” control 0 mM NaCl, (**i**) 100 mM NaCl, (**ii**) 200 mM NaCl, (**iii**) 300 mM NaCl).

The seeds of the selected landraces representing the four types of cultivated squash are illustrated in Figure 1. Regarding the different salt concentrations, landrace “746” (Batati orange) showed the highest germination percentage at all salt levels and was distinguished for its good tolerance to salt stress. Moreover, landrace “751” (Bejaoui green) expressed the lowest values especially at 200 and 300 mM, while landrace “748” (Kerkoubi orange) and “747” (Galaoui) could be also considered as similarly tolerant landraces and would be recommended for cultivation in saline soils or with irrigation water with a high salt level.

### 2.4. Effect of the Salt Stress Level on the Content of Malondialdehyde (MDA), Free Proline and Chlorophyll a and b

The results indicate that the different stress levels significantly affected the content of MDA, free proline and chlorophylls of squash seedlings (Table 5). Regarding MDA content, a significantly increased content was noted in stressed seedlings as compared to the control treatment (1.16 µmol mg^−1^ FW). The highest MDA content was recorded at 100 mM NaCl (1.75 µmol mg^−1^ FW), while the respective values for 200 and 300 mM NaCl did not differ significantly (1.43 and 1.34 µmol mg^−1^ FW). The content of free proline was the highest at the highest salinity level (0.92 µg mg^−1^ FW), whereas no significant differences were observed between the control and the 200 mM NaCl treatments (Table 5). An exception to this trend was noted at 100 mM NaCl, which showed the lowest values of free proline content (0.53 µg mg^−1^ FW). As expected, chlorophyll a (chla) and b (chlb) were reduced in stressed plants, as compared to the control treatment (Table 5), although the salt level of 100 mM NaCl showed a differential trend of increased chla and chlb (35.47 and 69.88 mg g^−1^ FW, respectively). The chlorophyll a values ranged from 25.70 to 35.47 mg g^−1^ FW, while chlorophyll b ranged from 52.44 to 69.88 mg g^−1^ FW. 

### 2.5. Effect of Landrace on the Content of Malondialdehyde, Free Proline and Chlorophyll a and b

The obtained results point to a significant effect of the landrace on the content of *MDA*, free proline and chlorophyll in squash seedlings (Table 6). In relation to MDA, the highest content was recorded in landrace “746” (1.60 µmol g^−1^ FW), while the values of all the other landraces did not differ significantly. The landrace “746” was further distinguished by the highest content of free proline (1.00 µg mg^−1^ FW). On the other hand, landrace “748” showed the lowest content of free proline (Table 6). With respect to chla and chlb, the lowest values were obtained in the landrace “746” (12.49 and 25.69 mg g^−1^ FW of chla and chlb, respectively), followed by “747” (14.81 and 27.89 mg g^−1^ FW of chla and chlb, respectively), whereas the highest content was noted in “751” (51.07 and 108.74 mg g^−1^ FW of chla and chlb, respectively) (Table 6).

### 2.6. Effect of Landrace and Salt Stress Level on the Content of Malondialdehyde, Free Proline and Chlorophyll a and b

Our findings indicate that the contents of MDA, proline and chlorophyll were differentially affected by the landrace and the salt stress level applied (Table 7). In relation to MDA, “748” landrace showed a decreasing trend as NaCl increased. Furthermore, all the other landraces exhibited a decreased content upon stress, yet they showed a notable increase either at 100 mM NaCl (“747” and “746”) or 300 mM NaCl (“751”). Accordingly, the response of these four landraces (“747”, “746”, “748” and “751”) to the different stress levels varied considerably in terms of the free proline content (Table 7). The stress response of most landraces involved an increased content of free proline, with the exception of landrace “746”, whose content was maximized in control plants. In relation to the stress level effects, the application of 200 mM NaCl induced proline accumulation in “748” and “751”, whereas “747” presented a peak at 300 mM NaCl (Table 7). Similarly, the content of chla and chlb was differentially affected both by the landrace and the stress level. Chlorophyll a and b were generally reduced in stressed plants, as compared to the control treatment, yet deviations from such a decreasing trend were observed depending on the landrace and stress intensity (Table 7). Specifically, “748” and “751” landraces showed the highest content of chla and chlb at 100 mM NaCl, while “747” presented the highest values at 300 mM NaCl. For the “746” landrace, the highest and lowest values for chla and chlb were noted at 0 mM and 200 mM NaCl, respectively. 

## 3. Discussion

The efficient selection of a salt-tolerant germplasm is considered of utmost importance in all breeding programs aimed at the development of salt-tolerant varieties. Given that salinity poses severe constraints to crop growth and productivity as well as to fruit quality in squash, this study aimed at determining the response of Tunisian squash germplasm to salinity stress at the stage of germination and early seedling growth. Plant response was assessed on the basis of selected traits related to seed germination and seedling growth potential. Based on their performance, four landraces were selected and further evaluated at the biochemical level, focusing on the determination of compounds that play a key role in the ability to withstand salt stress (e.g., MDA, free proline and chlorophylls). 

Our findings revealed that salinity stress substantially affects all traits associated with germination and early seedling growth, with the effects of stress being in most cases analogous to the stress level applied. Differences in germination potential were reflected at the final germination percentage, on the seventh day after salt stress initiation, as well as at the decrease in germination over the control treatment. These findings are consistent with previous reports regarding the effects of salt stress on the germination of various plant species, including lettuce [33,40], rice [41], chickpea [31] and wheat [42]. Indeed, plants’ sensitivity to salinity varies among growth stages [21], while during germination, the stress effects are aggravated due to the reduced seed water uptake that limits imbibition and seed turgescence [25]. Our data point to a gradual increasing severity of effects depending on stress intensity, thus supporting previous conclusions that the varying stress levels differently affect germination and seedling growth in various species, such as sugar beet and cabbage [43], soybean [44] and lentil [32]. Although all landraces were drastically affected, especially at high stress levels, their stress response varied significantly, thus indicating the existence of considerable genetic variation related to salt tolerance in the germplasm under study. As such, “746” and “747” were the best performing cultivars across stress levels, whereas “1007”, “1008” and “1009” landraces, despite their relatively high innate germinability, were incapable of germination at all stress levels. 

In addition to seed germination, the increasing level of salinity led to a gradually decreasing tissue elongation, expressed as reduced root and shoot lengths, probably as a result of toxicity as well as limited nutrient and water uptake due to osmotic stress [45,46]. Such data further support previous evidence that root and shoot lengths are the most suitable traits in terms of evaluating salt tolerance [31,47], since roots are responsible for water absorption and shoots for supplying aboveground tissues with water. In our study, seedling growth was inhibited at all salt stress levels, while root length was more severely affected than shoot length, most probably due to the fact that roots are directly and for a longer period exposed to salinity [46]. Indeed, such restricting effects have been attributed to the reduced water and nutrient absorption capacity from soil solution, which limits cell elongation and plant tissue development [48,49,50,51]. These findings are in agreement with the observed severe effects of salinity in roots of sugar beet, cabbage and amaranth [43] as well as soybean [44] and lentil [32]. Despite the fact that salinity led to a drastic reduction in seedling growth in all the tested landraces, a large variation between landraces was observed. At all stress levels studied, “1006” showed the highest SL and RL, therefore providing evidence for its salt tolerance ability, whereas the lowest values were noted in landraces “1007” and “1008” (in the case of SL) or “1008” (in the case of RL), indicating high susceptibility to salt stress. Accordingly, our data support a trend of gradually decreasing seedling fresh weight with increasing stress intensity, which could be mainly attributed to ionic effects occurring as a result of a proportional increase in Na+ concentration. Among landraces, “752” showed the highest growth potential as expressed by SFW and RFW parameters, both under normal and salt stress conditions, whereas “1008” and “747”, “1008” and “1009” were the most drastically affected in relation to SFW and RFW, respectively. Concomitant with the abovementioned findings, all the recorded physiological indices were severely affected by salinity stress, especially at high stress levels. The adverse effect of salt stress on GSTI, SLSTI and RLSTI has been previously evidenced in maize germplasm subjected to drought stress [52], while previous reports further underline the significant effect of the genotype on the response to salt stress for several crop species [43,51,53]. Our findings support the notion that stress effects are differentially expressed in the germplasm under study. As such, “747” proved to be the most superior landrace in terms of GSTI and SLSTI, while “1006” was characterized as the most tolerant landrace based on RLSTI. In contrast, the combined data of GSTI, SLSTI and RLSTI classified “1007”, “1008” and “1009” as the most salt-sensitive landraces. 

In addition to germination and seedling growth potential under stress conditions, an indicator routinely employed to assess the degree of damage to plant cells caused by abiotic stress is MDA accumulation—a natural product of lipid peroxidation due to oxidative stress [54,55,56]. Our data revealed a significant variation both in the landraces tested and the stress level applied. Although the mean MDA content across landraces was generally increased in stressed plants, as compared to the control treatment, such an increase was attributed to a peak in MDA content at specific stress levels. Interestingly, “746” and “747” showed the maximum content at 100 mM NaCl, while in landrace “751”, MDA content reached its peak at 300 mM NaCl. Such findings are in total agreement with the previously reported inconclusive differences in MDA level in purslane plants subjected to varying salt stress levels, where the MDA content showed a peak after exposure at 300 mM NaCl for 6 or 9 days [57]. In general, the increased MDA and O2- synthesis under salt stress has been associated with damage to cell membrane integrity and protein activity, although the damage recorded depends on the stress period [58]. It has been suggested that long-term exposure to high salinity may lead to the destruction of cell membranes, adversely affecting SOD, POD and CAT activities, whereas oxidative stress-mediated lipid peroxidation does not occur within a short period; e.g., up to 5 week exposure to salt stress [59]. Our data further point to inconclusive differences in free proline content at various stress levels. Although the response of most landraces to salt stress involved an increase in free proline, with the exception of “746”, the maximum content was observed either at 200 mM (“748” and “751”) or at 300 mM NaCl (“747”). Such observations further support the previously reported negative correlation between MDA and free proline content [54], yet they partly deviate from the suggested positive association between proline content and stress intensity [57]. In our study, this association was only evidenced in landrace “747”, whose overall performance classified it as salt-tolerant, thus supporting previous conclusions related to the increased proline content in salt-tolerant genotypes of various plant species, including potato [60], melon [61] and tomato [62]. It is well evidenced that plants’ response to high salinity adversely affects photosynthesis activity, thus involving a reduction in chlorophyll content. Our data showed a general decreasing trend of chla and chlb in stressed plants, as compared to the control treatment, which indicates that the response to high salinity may involve a decrease in chlorophyll content in various plant species, including pepper [63] and winter squash [64]. However, deviations from this trend were noted among the tested landraces and the stress levels applied, indicating a genotype-depended response. Interestingly, the content of both chla and chlb was the maximum at 100 mM NaCl across landraces, with this peak being mostly evidenced in landraces “748” and “751”, thus probably reflecting an adaptive stress response governing tolerance to low-medium salinity. 

Overall findings provide conclusive evidence that the evaluation of squash genotypes at germination phase shows a great potential for revealing genetic variability related to salt tolerance. Addressing the classification of genotypes in terms of salt tolerance, our data related to germination and seedling growth potential under salt stress point to the superiority of landraces “746” and “747”, followed by “1006”, at all stress levels applied. In contrast, “1007”, “1008” and “1009” landraces were incapable of germination at all stress levels, thus proving their sensitivity to salinity even at relatively low stress levels. Our findings further support previous reports highlighting that the innate genotypic high growth potential is associated with salt sensitivity and vice versa [65]. Mostly relevant to this argument is the performance of landrace “751”: although it exhibited an enhanced seedling growth under normal conditions, it suffered a significant decrease after exposure to salt stress. Addressing the evaluation of salt tolerance based on biochemical parameters, the observed cumulative patterns for MDA, free proline and chla and chlb deviate considerably from the general stress response profiles that usually involve an increased MDA and proline content as well as a decreasing chlorophyll content with increasing salt concentration. Despite such deviations, which further strengthen previous reports on inconclusive differences in MDA and chlorophyll content in various plant species subjected to salt stress [57,65,66], the overall data support the feasibility of conducting the early selection of salt-tolerant squash germplasm on the basis of germination and seedling growth potential under salt stress.

## 4. Materials and Methods

### 4.1. Plant Material and Growth Conditions

The studied genetic material consisted of 15 Tunisian squash landraces, collected from different geographic regions of Tunisia during the period extending from 2018 to 2020 (Table 8). Each landrace was assigned passport data and an inventory number, according to the National Gene Bank of Tunisia, while full details are available at the Germplasm Resources Information Network—GRIN (http://www.tn-grin.nat.tn/gringlobal/search.aspx, accessed on 15 February 2022). The description of fruit morphology was performed based on the European Cooperative Program for Plant Genetic Resources (ECPGR) list of descriptors for *Cucurbita* spp. [67].

### 4.2. Salinity Stress Treatments 

The experiment was carried out at the Department of Horticulture, High Agronomic Institute of Chott Mariem-Sousse-Tunisia. Following the selection of seeds for size homogeneity, 50 seeds per landrace (five petri dishes with 10 seeds each, *n* = 5) were surface-sterilized for 5 min in 10% H_2_O_2_ (*v*/*v*) and rinsed twice in sterile dH_2_O. Sterilized seeds were primed via exposure to an eliciting solution of 1.5 mM gibberellic acid (GA_3_) for 24 h to stimulate germination and subsequently rinsed in sterile dH_2_O. Five to ten seeds, according to size, were placed on sterile petri dishes containing two layers of filter paper moistened daily with 5 mL of appropriate solutions: dH_2_O (control), 100, 200 and 300 mM NaCl. Seedlings were grown under controlled conditions for 7 days (25 ± 2 °C, 50 ± 5% relative humidity, 18 h light/6 h dark photoperiod under white fluorescent light (40 µmol m^−2^ s^−1^).

### 4.3. Determination of Germination and Seedling Growth Potential under Salt Stress

Salt tolerance was evaluated on the basis of various parameters related to seed germination and seedling growth potential under salt stress conditions, measured daily until no more germinated seeds were recorded (first–seventh day) (Table 9). Seeds were considered germinated when the protruding radicle was at least 2 mm long. The parameters germination reduction (GR), root length reduction (RLR) and shoot length stress tolerance index (SLSTI) express the decreased values of salt-stressed plants over the control treatment.

### 4.4. Evaluation of Salinity Tolerance Based on Biochemical Parameters

Based on the obtained data related to the response of the 15 landraces to salinity stress, 4 landraces representing the main types of cultivated squash were selected for further evaluation. As such, the landraces “748” (Karkoubi), “751” (Bejaoui), “747” (Galaoui) and “746” (Batati) were assessed, employing the content of seedling tissues in MDA (MDA), free proline and chlorophyll a and b as evaluation criteria.

The content of MDA (MDA) was determined using the method applied by Hnilickova et al. [57] with minor modifications. Briefly, 200 mg of leaf samples were homogenized with liquid nitrogen and, following the addition of 80% ethanol, samples were centrifuged for 5 min at 6000 rpm. Aliquots of 0.7 mL of the supernatant solution were mixed with 0.7 mL of 0.65% thiobarbituric acid (TBA) in 20% TCA (trichloroacetic acid) and 0.01% BHT (butylated hydroxytoluene). A second set of 0.7 mL samples was mixed with 0.7 mL of 20 % TCA and 0.01 % BHT. Following incubation at 95 °C for 25 min and subsequent cooling, samples were centrifuged for 5 min at 6000 rpm. The content of MDA was determined at 532 nm using a UV–Vis spectrophotometer (Evolution 210, Thermo Scientific, Abingdon, UK) and expressed in µmol g^−1^ of fresh weight (FW).

The content of free proline was measured according to the method described by Monneveux and Nemmar [68]. Leaf samples (100 mg) were homogenized in 10 mL of 3% sulfosalicylic acid and, following filtration, the homogenate was heated to 85 °C in a water bath for 60 min. After cooling, 1 mL of ninhydrin reagent was added (ninhydrin reagent consisted of 120 mL distilled water, 300 mL of acetic acid, 80 mL acetic orthophosphoric acid at a density of 1.7, and 25 mg of ninhydrin). The samples were boiled for 30 min and, after cooling, 5 mL of toluene was added and samples were vortexed. The upper phase was recovered and was measured using a UV-Vis spectrophotometer (Evolution 210, Thermo Scientific, Abingdon, UK) at 528 nm. A proline standard curve ranging from 0 to 2.5 mg mL^−1^ of L-proline was used to determine the proline content, expressed in µg mg^−1^ of FW.

For chlorophyll content determination, the extraction of samples was performed as described by Curtis and Shetty [69]. Briefly, 50 mg of leaf tissue (in triplicate) was extracted into 3 mL of methanol and stored at 23 °C in darkness for 2 h. Absorption of extracts (1.5 mL) was measured at 650 and 665 nm using a spectrophotometer (Evolution 210, Thermo Scientific, Abingdon, UK). Chlorophyll a and chlorophyll b were expressed in mg g^−1^ FW.

### 4.5. Statistical Analysis

The experimental layout was completely randomized with three replications. Data were analyzed using ANOVA tests (*p* ≤ 0.05), according to the experimental design, combining salt concentrations and genotypes. Differences between means were compared using the Duncan Multiple Range test (DMRT). Statistical analyses were performed using SAS software V9 (SAS Institute, Cary, CA, USA).

## 5. Conclusions

Soil salinization is gradually increasing due to the scarcity of rains and the increase in evapotranspiration, adversely affecting plant germination, growth, development and fruit setting in salt-affected soils, as is the case of semiarid regions. Most vegetable species are salt-sensitive at all stages of their lifecycle, while germination and seedling growth have been viewed as the critical stage under salt conditions. In particular, salinity is a factor that severely limits squash growth and productivity while, at the same time, deteriorating fruit quality. Despite the scientific evidence in relation to the salt stress response in a plethora of plant species, there is a lack of information in relevant research fields for squash. The development of a salt-tolerant germplasm is one of the most effective means for enhancing squash production in saline soils. The results of our study suggest the feasibility of conducting an early selection of salt-tolerant squash germplasm on the basis of germination and seedling growth potential under salt stress. Such an approach may considerably upgrade all procedures aimed at selecting salt-tolerant germplasm to be exploited for cultivation in saline soils. In this context, landraces “746” and “747” were the best performing cultivars for the tested salinity levels in terms of germination percentage, germination percentage stress index and shoot length stress index, which indicates that they could be integrated as valuable germplasm material in breeding programs targeted at improving salt tolerance in squash through the selection of elite genotypes.

## Figures and Tables

**Table 1:** Analysis of variance (mean of squares) for traits related to seed germination and seeding growth in squash germplasm (accession) under different salt stress conditions (Salinity).

S.O.V.	DF	GP	SL	RL	SFW	RFW	SRR	GR	SLR	RLR	GSTI	SLSTI	RLSTI
Accession	14	4066.8	341.0	152.9	2.6	0.05	54.8	3801.8	487.9	115.3	0.39	26,109.5	18,310.1
Salinity	3	17,405.8	1103.6	697.3	8.4	0.25	139.3	7553.0	22.9	227.9	0.98	1484.6	44,510.7
Accession x Salinity	42	620.4	46.7	14.1	0.2	0.004	18.9	455.4	9.1	6.6	0.05	669.1	1321.4
CV (%)		13.3	9.0	11.3	17.0	24.9	20.6	10.2	20.7	21.7	29.69	15.4	16.6

S.O.V.: source of variance; DF: degree of freedom. CV: coefficient of variance; GP: germination percentage; SL: shoot length: RL: root length; SFW: shoot fresh weight; RFW: root fresh weight; SRR: shoot length/root length ratio; GR: germination reduction; SLR: shoot length reduction; RLR: root length reduction; GSTI: germination stress tolerance index; SLSTI: shoot length stress tolerance index; RLSTI: root length stress tolerance index.

**Table 2:** Mean effect of the salt stress level (0, 100, 200 and 300 mM NaCl) on germination potential and seedlings characteristics (mean ± SD), regardless of the squash accession.

NaCl Concentration (mM)	GP (%)	SL (mm)	RL (mm)	SFW (g)	RFW (g)	SRR	GR (%)	SLR (%)	RRL (%)	GSTI (%)	SLSTI (%)	RLSTI (%)
Control	86.3 ± 1.08 a	12.3 ± 0.24 a	7.4 ± 0.18 a	0.9 ± 0.02 a	0.14 a ± 0.004 a	1.3 ± 0.02 c	-	-	-	-	-	-
100	23.3 ± 1.50 b	7.1 ± 0.33 b	4.7 ± 0.24 b	0.6 ± 0.03 b	0.08 ± 0.004 b	1.7 ± 0.06 b	63.0 ± 1.49 c	5.2 ± 0.37 b	2.7 ± 0.22 c	0.27 ± 0.016 a	60.1 ± 2.83 a	63.8 ± 2.94 a
200	15.0 ± 1.08 c	6.4 ± 0.31 c	3.3 ± 0.20 c	0.5 ± 0.05 c	0.06 ± 0.003 c	1.8 ± 0.10 b	71.3 ± 1.18 b	5.9 ± 0.35 a	4.1 ± 0.19 b	0.17 ± 0.011 b	54.2 ± 2.58 b	44.0 ± 2.34 b
300	8.4 ± 0.67 d	6.3 ± 0.30 c	2.0 ± 0.14 d	0.3 ± 0.01 d	0.04 ± 0.003 d	3.6 ± 0.27 a	77.9 a ± 1.02 a	5.9 ± 0.39 a	5.3 ± 0.16 a	0.09 ± 0.006 v	54.6 ± 2.84 b	27.5 ± 1.77 c
F Value	8816 **	2141.20 **	2879.11 **	815.17 **	649.38 **	745.38 **	362.56 **	143.95 **	791.03 **	296.89 **	19.75 **	16.58 **

** Means in the same column followed by the same letter are not significantly different at *p* < 0.05, according to Duncan’s Multiple Range test; Parameters from GR to RLSTI were evaluated for all landraces compared to the control (see material and methods); GP: germination percentage; SL: shoot length: RL: root length; SFW: shoot fresh weight; RFW: root fresh weight; SRR: shoot length/root length ratio; GR: germination reduction; SLR: shoot length reduction; RLR: root length reduction; GSTI: germination stress tolerance index; SLSTI: shoot length stress tolerance index; RLSTI: root length stress tolerance index.

**Table 3:** The mean effect of the squash germplasm accessions on traits related to seed germination and seedling growth (mean ± SD), regardless of the salinity level (NaCl concentration).

Accession	GP (%)	SL (mm)	RL (mm)	SFW (g)	RFW (g)	SRR	GR (%)	SLR (%)	RRL (%)	GSTI (%)	SLSTI (%)	RLSTI (%)
“745”	42.0 ± 4.72 bc	8.0 ± 0.18 gh	4.7 ± 0.29 de	0.6 ± 0.02 de	0.07 ± 0.008 e	1.8 ± 0.58 h	74.6 ± 3.55 c	6.0 ± 1.11 d	2.0 ± 0.56 h	0.24 ± 0.04 e	56.67 ± 7.45 e	68.9 ± 7.47 b
“746”	48.4 ± 5.36 a	8.3 ± 0.32 g	4.5 ± 0.24 e	0.6 ± 0.007 de	0.02 ± 0.005 h	2.4 ± 0.26 e	67.0 ± 3.15 de	2.1 ± 0.69 h	3.6 ± 0.74 e	0.32 ± 0.03 ab	80.85 ± 5.66 a	49.8 ± 7.55 e
“747”	49.3 ± 4.81 a	7.8 ± 0.16 h	3.0 ± 0.26 g	0.6 ± 0.03 de	0.03 ± 0.005 fg	3.6 ± 0.36 b	64.0 ± 2.64 ef	1.6 ± 0.25 h	2.5 ± 0.61 fh	0.34 ± 0.07 a	82.25 ± 7.23 a	44.3 ± 8.50 ef
“748”	41.5 ± 7.02 cd	9.3 ± 1.11 d	4.7 ± 0.66 de	0.8 ± 0.05 b	0.10 ± 0.008 cd	2.1 ± 0.72 f	64.6 ± 2.49 ef	4.8 ± 0.89 e	2.0 ± 0.55 gh	0.29 ± 0.02 cd	64.20 ± 5.15 d	67.6 ± 8.47 b
“749”	40.0 ± 4.93 cd	9.3 ± 0.29 de	4.0 ± 0.41 f	0.6 ef ± 0.01 ef	0.10 ± 0.006 cd	2.9 ± 0.32 d	60.0 ± 3.56 g	4.1 ± 0.81 f	4.1 ± 0.64 d	0.30 ± 0.02 bc	72.54 ± 6.80 b	42.9 ± 7.89 gh
“750”	39.5 ± 5.05 d	5.3 ± 0.81 i	3.0 ± 0.51 g	0.8 ± 0.05 c	0.10 ± 0.006 cd	3.3 ± 0.24 c	71.3 ± 4.40 c	2.0 ± 0.66 h	2.8 ± 0.52 f	0.23 ± 0.03 e	73.00 ± 6.77 b	44.7 ± 8.12 fg
“751”	31.2 ± 4.83 e	9.8 ± 0.35 c	8.0 ± 0.36 b	0.9 ± 0.02 b	0.09 ± 0.004 d	1.2 ± 0.23 i	65.0 ± 5.06 ef	10.6 ± 1.35 c	5.3 ± 0.79 c	0.19 ± 0.05 f	40.54 ± 5.44 f	57.4 ± 7.36 c
“752”	42.7 ± 4.10 b	8.8 ± 0.37 f	5.8 ± 0.36 c	1.1 ± 0.03 a	0.15 ± 0.006 a	1.5 ± 0.34 h	71.0 ± 2.64 cd	3.8 ± 0.85 f	2.3 ± 0.50 gh	0.26 ± 0.02 de	67.43 ± 6.70 cd	70.5 ± 5.47 b
“753”	28.7 ± 5.47 f	9.0 ± 0.24 ef	3.0 ± 0.31 g	0.6 ± 0.05 d	0.11 ± 0.005 b	4.5 ± 0.52 a	61.7 ± 4.04 fg	3.0 ± 0.80 h	3.4 ± 0.71 e	0.18 ± 0.04 f	81.11 ± 8.30 a	32.6 ± 4.55 h
“1004”	23.2 ± 5.09 gh	10.9 ± 0.24 b	4.7 ± 0.57 d	0.5 ± 0.02 f	0.10 ± 0.006 cd	3.1 ± 0.21 c	66.3 ± 3.20 e	3.6 ± 0.84 f	4.1 ± 0.82 d	0.09 ± 0.03 h	73.98 ± 6.21 b	50.8 ± 8.24 de
“1005”	22.0 ± 3.23 gh	10.7 ± 0.22 b	5.9 ± 0.62 c	0.6 ± 0.04 de	0.10 ± 0.006 cd	1.9 ± 0.18 fg	54.7 ± 6.80 h	4.2 ± 0.91 f	4.2 ± 0.80 d	0.13 ± 0.20 g	69.99 ± 5.41 bc	54.6 ± 4.91 cd
“1006”	28.1 ± 4.95 f	13.4 ± 0.85 a	8.3 ± 0.38 a	0.4 ± 0.03 g	0.10 ± 0.006 cd	1.7 ± 0.16 gh	92.3 ± 2.77 a	2.8 ± 0.60 g	2.1 ± 0.58 gh	0.15 ± 0.01 i	81.98 ± 7.16 a	81.6 ± 7.93 a
“1007”	21.5 ± 6.76 h	2.7 ± 0.34 k	1.8 ± 0.30 i	0.2 ± 0.03 h	0.04 ± 0.007 f	0.4 ± 0.10 j	86.0 ± 2.58 b	10.7 ± 1.42 c	7.4 ± 0.88 b	0.03 ± 0.01 i	0.00 ± 0.00 g	0.0 ± 0.00 i
“1008”	16.7 ± 6.30 i	2.9 ± 0.78 k	1.3 ± 0.54 j	0.1 ± 0.06 i	0.03 ± 0.001 h	0.5 ± 0.13 j	67.0 ± 4.99 de	11.6 ± 1.54 b	5.3 ± 0.84 c	0.04 ± 0.01 i	0.00 ± 0.00 g	0.0 ± 0.00 i
“1009”	24.0 ± 4.94 g	3.8 ± 0.38 j	2.3 ± 0.21 h	0.2 ± 0.04 h	0.03 ± 0.006 h	0.4 ± 0.10 j	96.0 ± 4.40 a	15.1 ± 1.66 a	9.1 ± 0.96 a	0.03 ± 0.01 i	0.00 ± 0.00 g	0.0 ± 0.00 i
CV %	13.54	8.96	11.32	16.98	23.92	20.63	10.23	20.69	21.74	26.69	15.39	16.62

Means in the same column followed by the same letter are not significantly different at *p* < 0.05, according to Duncan’s multiple range test; GP: germination percentage; SL: shoot length: RL: root length; SFW: shoot fresh weight; RFW: root fresh weight; SRR: shoot length/root length ratio; GR: germination reduction; SLR: shoot length reduction; RLR: root length reduction; GSTI: germination stress tolerance index; SLSTI: shoot length stress tolerance index; RLSTI: root length stress tolerance index.

**Table 4:** Response of squash germplasm accessions to varying salt stress levels (0, 100, 200 and 300 mM NaCl) in relation to traits (means ± SD) related to seed germination and seedling growth.

NaCl Concentration (mM)	Accession	GP (%)	SL (mm)	RL (mm)	SFW (g)	RFW (g)	SRR
Control	“745”	97.9 ± 1.5	12.5 ± 1.6	6.2 ± 0.8	0.9 ± 0.2	0.15 ± 0.02	2.0 ± 0.4
	“746”	98.7 ± 1.5	9.9 ± 1.8	7.2 ± 0.7	0.8 ± 0.1	0.04 ± 0.01	1.4 ± 0.2
	“747”	97.3 ± 2.3	9.1 ± 0.7	4.9 ± 0.5	0.7 ± 0.4	0.07 ± 0.03	1.9 ± 0.2
	“748”	90.0 ± 8.7	12.9 ± 1.8	6.2 ± 0.6	1.1 ± 0.3	0.15 ± 0.02	2.1 ± 0.4
	“749”	85.0 ± 4.3	12.4 ± 1.6	7.0 ± 1.2	0.76 ± 0.06	0.15 ± 0.01	1.8 ± 0.4
	“750”	93.0 ± 2.6	6.8 ± 0.7	5.1 ± 0.3	1.1 ± 0.1	0.16 ± 0.02	1.3 ± 0.2
	“751”	80.0 ± 4.3	17.8 ± 1.8	12.0 ± 2.0	1.3 ± 0.2	0.14 ± 0.02	1.5 ± 0.1
	“752”	96.0 ± 2.6	11.7 ± 1.1	7.5 ± 0.6	1.7 ± 0.2	0.19 ± 0.05	1.4 ± 0.2
	“753”	75.0 ± 4.3	10.4 ± 0.4	5.6 ± 0.3	0.83 ± 0.03	0.18 ± 0.02	1.9 ± 0.1
	“1004”	73.0 ± 5.7	13.5 ± 1.1	7.7 ± 1.4	0.63 ± 0.05	0.15 ± 0.01	1.8 ± 0.3
	“1005”	63.0 ± 6.7	13.9 ± 1.0	9.1 ± 1.1	0.8 ± 0.1	0.15 ± 0.03	1.5 ± 0.2
	“1006”	97.3 ± 1.9	15.5 ± 0.5	9.9 ± 0.4	0.69 ± 0.07	0.16 ± 0.02	1.6 ± 0.1
	“1007”	86.0 ± 5.2	10.7 ± 0.4	7.4 ± 0.4	0.86 ± 0.03	0.17 ± 0.02	1.4 ± 0.1
	“1008”	67.0 ± 5.7	11.6 ± 0.8	5.3 ± 0.3	0.52 ± 0.03	0.16 ± 0.02	2.2 ± 0.2
	“1009”	96.0 ± 2.6	15.1 ± 0.8	9.1 ± 0.3	0.73 ± 0.09	0.10 ± 0.01	1.7 ± 0.1
100	“745”	50.0 ± 5.7	7.7 ± 0.6	5.0 ± 0.5	0.7 ± 0.04	0.08 ± 0.01	1.5 ± 0.2
	“746”	40.0 ± 4.3	9.0 ± 0.6	5.7 ± 0.5	0.6 ± 0.2	0.03 ± 0.05	1.6 ± 0.2
	“747”	45.0 ± 4.3	8.0 ± 0.4	3.8 ± 0.4	0.68 ± 0.04	0.04 ± 0.01	2.1 ± 0.2
	“748”	35.0 ± 3.3	8.6 ± 0.5	5.3 ± 0.2	0.9 ± 0.2	0.10 ± 0.03	1.6 ± 0.1
	“749”	40.0 ± 4.8	9.0 ± 0.9	4.4 ± 0.3	0.64 ± 0.03	0.10 ± 0.01	2.0 ± 0.3
	“750”	30.0 ± 5.3	4.8 ± 0.9	4.5 ± 0.3	0.92 ± 0.06	0.11 ± 0.01	1.1 ± 0.2
	“751”	30.0 ± 5.3	8.5 ± 0.5	9.3 ± 0.3	1.00 ± 0.04	0.11 ± 0.01	0.9 ± 0.1
	“752”	35.0 ± 4.3	8.9 ± 0.5	6.3 ± 0.3	1.29 ± 0.08	0.17 ± 0.01	1.4 ± 0.1
	“753”	25.0 ± 3.3	7.6 ± 0.4	3.5 ± 0.3	0.71 ± 0.04	0.12 ± 0.01	2.2 ± 0.2
	“1004”	25.0 ± 3.3	10.4 ± 0.7	6.2 ± 0.3	0.58 ± 0.02	0.11 ± 0.01	1.7 ± 0.1
	“1005”	15.0 ± 2.6	11.1 ± 0.8	6.5 ± 0.2	0.69 ± 0.02	0.10 ± 0.01	1.7 ± 0.2
	“1006”	10.0 ± 4.4	12.2 ± 0.5	9.4 ± 0.3	0.44 ± 0.03	0.10 ± 0.01	1.3 ± 0.1
	“1007”	0	0	0	0	0	0
	“1008”	0	0	0	0	0	0
	“1009”	0	0	0	0	0	0
200	“745”	15.0 ± 4.3	6.6 ± 0.4	4.1 ± 0.2	0.53 ± 0.03	0.04 ± 0.00	1.6 ± 0.1
	“746”	35.0 ± 4.3	7.7 ± 0.6	3.4 ± 0.3	0.50 ± 0.04	0.01 ± 0.00	2.3 ± 0.3
	“747”	35.0 ± 4.3	7.5 ± 0.4	2.2 ± 0.3	0.55 ± 0.03	0.01 ± 0.00	3.5 ± 0.5
	“748”	25.0 ± 3.2	8.2 ± 0.3	4.1 ± 0.2	0.74 ± 0.03	0.08 ± 0.03	2.0 ± 0.1
	“749”	25.0 ± 3.2	8.2 ± 0.7	3.0 ± 0.3	0.49 ± 0.14	0.08 ± 0.01	2.8 ± 0.3
	“750”	25.0 ± 3.2	3.7 ± 0.5	1.6 ± 0.2	0.64 ± 0.02	0.08 ± 0.01	2.3 ± 0.3
	“751”	10.0 ± 4.2	7.2 ± 0.4	6.4 ± 0.3	0.85 ± 0.04	0.08 ± 0.01	1.1 ± 0.1
	“752”	25.0 ± 3.3	7.6 ± 0.4	5.9 ± 0.3	0.88 ± 0.03	0.13 ± 0.01	1.3 ± 0.1
	“753”	10.0 ± 3.3	8.3 ± 0.3	2.2 ± 0.2	0.57 ± 0.03	0.09 ± 0.03	3.9 ± 0.3
	“1004”	5.0 ± 1.7	9.8 ± 0.7	3.4 ± 0.3	0.53 ± 0.02	0.08 ± 0.01	2.9 ± 0.4
	“1005”	10.0 ± 4.2	9.8 ± 0.3	4.7 ± 0.1	0.51 ± 0.03	0.08 ± 0.01	2.1 ± 0.1
	“1006”	5.0 ± 1.7	10.8 ± 0.5	8.5 ± 0.4	0.33 ± 0.02	0.08 ± 0.01	1.3 ± 0.1
	“1007”	0	0	0	0	0	0
	“1008”	0	0	0	0	0	0
	“1009”	0	0	0	0	0	0
300	“745”	5.0 ± 1.7	5.3 ± 0.6	3.5 ± 0.3	0.33 ± 0.03	0.02 ± 0.00	1.5 ± 0.2
	“746”	20.0 ± 3.3	6.7 ± 0.7	1.6 ± 0.3	0.50 ± 0.01	0.01 ± 0.00	4.0 ± 0.9
	“747”	25.0 ± 3.3	6.7 ± 0.4	1.0 ± 0.2	0.47 ± 0.03	0.01 ± 0.00	7.0 ± 1.4
	“748”	17.0 ± 4.1	7.7 ± 0.3	3.0 ± 0.3	0.5 ± 0.1	0.07 ± 0.01	2.6 ± 0.2
	“749”	10.0 ± 3.2	7.5 ± 0.5	1.6 ± 0.2	0.35 ± 0.03	0.05 ± 0.01	5.0 ± 1.0
	“750”	10.0 ± 3.2	6.0 ± 1.0	0.7 ± 0.1	0.32 ± 0.04	0.06 ± 0.02	8.5 ± 1.3
	“751”	5.0 ± 1.7	5.6 ± 0.6	4.4 ± 0.3	0.35 ± 0.03	0.04 ± 0.01	1.3 ± 0.1
	“752”	15.0 ± 4.3	6.9 ± 0.4	3.6 ± 0.2	0.64 ± 0.03	0.12 ± 0.01	1.9 ± 0.1
	“753”	5.0 ± 1.7	9.4 ± 0.3	0.9 ± 0.1	0.38 ± 0.02	0.05 ± 0.02	10.3 ± 1.5
	“1004”	5.0 ± 1.7	9.8 ± 0.7	1.6 ± 0.2	0.35 ± 0.02	0.05 ± 0.01	6.2 ± 0.8
	“1005”	5.0 ± 1.7	8.1 ± 0.5	3.5 ± 0.2	0.3 ± 0.1	0.05 ± 0.03	2.3 ± 0.1
	“1006”	5.0 ± 1.7	15.2 ± 0.5	5.4 ± 0.3	0.21 ± 0.02	0.06 ± 0.02	2.8 ± 0.1
	“1007”	0	0	0	0	0	0
	“1008”	0	0	0	0	0	0
	“1009”	0	0	0	0	0	0

GP: germination percentage; SL: shoot length: RL: root length; SFW: shoot fresh weight; RFW: root fresh weight; SRR: shoot length/root length ratio.

**Table 5:** Mean effect of the salt stress level (0, 100, 200 and 300 mM NaCl) on the content of malondialdehyde (MDA), free proline and chlorophyll a and b (mean ± SD), regardless of the pumpkin accession.

NaCl concentration (mM)	MDA (µmol g^−1^)	Proline (µg mg^−1^)	Chl a (mg mg^−1^)	Chl b (mg mg^−1^)
Control	1.17 ± 0.53 b	0.82 ± 0.06 a	30.09 ± 2.87 b	60.64 ± 5.96 b
100	1.75 ± 0.30 a	0.51 ± 0.02 b	35.47 ± 3.90 a	69.88 ± 7.68 a
200	1.43 ± 0.21 b	0.82 ± 0.04 a	25.70 ± 2.43 d	52.44 ± 5.50 d
300	1.34 ± 0.18 b	0.92 ± 0.05 a	28.91 ± 2.46 c	56.34 ± 5.98 c
F Value	33.6 **	471.2 **	552.3 **	737.5 **
SD				

** Means in the same column followed by the same letter are not significantly different at *p* < 0.05, according to Duncan’s multiple range test.

**Table 6:** Mean effect of the squash germplasm accession on the content of malondialdehyde (MDA), free proline and chlorophyll a and b (mean ± SD), regardless of the salinity level (NaCl concentration).

Accession	MDA (µmol g^−1^)	Proline (µg mg^−1^)	Chl a (mg mg^−1^)	Chl b (mg mg^−1^)
“746”	1.6 ± 0.12 a	1.0 ± 0.06 a	12.5 ± 1.50 d	25.7 ± 2.08 d
“747”	0.8 ± 0.19 b	0.8 ± 0.02 b	14.8 ± 1.16 c	27.9 ± 2.15 c
“748”	0.8 ± 0.12 b	0.5 ± 0.04 d	41.8 ± 1.50 b	77.0 ± 2.08 b
“751”	0.9 ± 0.26 b	0.6 ± 0.05 c	51.1 ± 1.29 a	108.7 ± 3.82 a
F Value	15.5 **	1466.3 **	12473.9 **	21337.9 **
SD				

** Means in the same column followed by the same letter are not significantly different at *p* < 0.05, according to Duncan’s multiple range test.

**Table 7:** Response of squash germplasm accession to varying salt stress levels (0, 100, 200 and 300 mM NaCl) in relation to the content of malondialdehyde (MDA), free proline and chlorophyll a and b (mean ± SD).

Accession	NaCl Concentration (mM)	MDA (µmol g^−1^)	Proline (µg mg^−1^)	Chl a (mg mg^−1^)	Chl b (mg mg^−1^)
“746”	Control	0.88 ± 0.03	1.42 ± 0.03	14.7 ± 0.2	31.1 ± 0.7
	100	4.24 ± 0.03	0.74 ± 0.01	11.6 ± 0.2	24.4 ± 0.5
	200	0.58 ± 0.08	1.13 ± 0.05	9.9 ± 0.2	20.7 ± 0.3
	300	0.68 ± 0.09	0.71 ± 0.08	13.7 ± 0.1	26.6 ± 0.8
“747”	Control	0.9 ± 0.1	0.59 ± 0.02	13.7 ± 0.1	26.0 ± 0.3
	100	1.9 ± 0.6	0.44 ± 0.01	13.2 ± 0.2	25.8 ± 0.2
	200	0.36 ± 0.06	0.56 ± 0.02	15.6 ± 0.4	29.5 ± 0.5
	300	0.5 ± 0.2	0.8 ± 0.009	16.8 ± 0.4	30.2 ± 1.1
“748”	Control	1.8 ± 0.4	0.47 ± 0.05	37.5 ± 0.6	72.2 ± 0.9
	100	0.65 ± 0.03	0.48 ± 0.04	57.0 ± 0.8	103.6 ± 1.5
	200	0.60 ± 0.04	0.55 ± 0.05	30.2 ± 0.6	55.2 ± 0.6
	300	0.20 ± 0.07	0.40 ± 0.01	42.5 ± 0.8	76.9 ± 0.9
“751”	Control	1.1 ± 0.2	0.63 ± 0.09	54.5 ± 0.5	113.3 ± 1.7
	100	0.2 ± 0.1	0.46 ± 0.01	60.0 ± 3.8	125.7 ± 3.9
	200	0.20 ± 0.03	1.06 ± 0.05	47.1 ± 0.4	104.3 ± 3.5
	300	1.56 ± 0.20	0.98 ± 0.03	42.7 ± 0.3	91.7 ± 2.4

**Table 8:** Description of the Tunisian squash landraces employed in this study.

Landrace Inventory Number	Local Name	Origin	Latitude	Longitude	Short Description
NGBTUN745 (“745”)	Batati Green	Ariana (Kalaat Andalous)	37°033″ N	10°11′7″ E	Globular fruit, light green skin, green flesh
NGBTUN746 (“746”)	Batati orange	Siliana (SidiHamada)	35°57′28″ N	9°32′57″ E	Globular fruit, orange skin, light orange flesh
NGBTUN747 (“747”)	Galaoui	Ariana (Kalaa Andalous)	37°033″ N	10°11′7″ E	Raised fruit with basal tip, green skin, green flesh
NGBTUN748 (“748”)	Karkoubi orange	Sousse (SidiBouali)	35°54′22.21″ N	10°32′47.81″ E	Flattened fruit, dark yellow skin, yellow flesh
NGBTUN749 (“749”)	Batati yellow spotted with white	Siliana (SidiHamada)	35°57′28″ N	9°32′57″ E	Globular fruit, orange skin spotted with white, orange flesh
NGBTUN750 (“750”)	Batati white	Monastir (Sahline)	35°45′05″ N	10°42′39″ E	Globular fruit, white skin, white flesh
NGBTUN751 (“751”)	Bejaoui Green	Siliana (SidiHamada)	35°57′28″ N	9°32′57″ E	Flattened fruit, dark green skin, light green flesh
NGBTUN752 (“752”)	Batati yellow	Siliana (North)	35°57′28″ N	9°32′57″ E	Globular fruit, yellow skin, light orange flesh
NGBTUN753 (“753”)	Béjaoui Green	Siliana (South)	35°57′28″ N	9°32′57″ E	Flattened fruit, dark green skin, light green flesh
NGBTUN1004 (“1004”)	Galaoui large seeds	Ariana (Kalaat Andalous)	37°033″ N	10°11′7″ E	Turbinate interior fruit with basal tip, green skin, white green flesh
NGBTUN1005 (“1005”)	Galaoui smoll seeds	Ariana (Kalaat Andalous)	37°033″ N	10°11′7″ E	Turbinate interior fruit with a big basal tip, green skin, white green flesh
NGBTUN1006 (“1006”)	Karkoubi orange	Monastir (Sahline)	35°45′05″ N	10°42′39″ E	Flattened fruit, dark yellow skin, yellow flesh
NGBTUN1007 (“1007”)	Batati Green	Siliana	35°57′28″ N	9°32′57″ E	Rounded fruit, green skin, green flesh
NGBTUN1008 (“1008”)	Batati Green	Monastir (Teboulba)	35°45′05″ N	10°42′39″ E	Globular fruit, flat stem end, green skin, light green flesh
NGBTUN1009 (“1009”)	Bejaoui spotted with yellow	Siliana (SidiHamada)	35°57′28″ N	9°32′57″ E	Globular fruit with flat stem end, spotted with yellow light green skin, light green flesh

**Table 9:** Description of the evaluation criteria for salinity tolerance employed in this study.

Trait	Unit	Description/Formula	Reference
Germination percentage (GP)	%	$\mathrm{GP}=\frac{\mathrm{number}\;\mathrm{germinated}\;\mathrm{seeds}}{\mathrm{number}\;\mathrm{of}\;\mathrm{total}\;\mathrm{seeds}}\times 100$	Scott et al. (1984)
Shoot length (SL)	mm	At the day of germination	Sivakumar et al. (2020)
Root length (RL)	mm	At the day of germination	Sivakumar et al. (2020)
Shoot fresh weight (SFW)	g	Recorded by using a sensitive balance (Sartorius AC 1215, Germany)	Jamil et al. (2006)
Root fresh weight (RFW)	g	Recorded by using a sensitive balance (Sartorius AC 1215, Germany)	Jamil et al. (2006)
Shoot length/Root length Ratio (SRR)	-	Ratio of SL to RL	Thabet et al. (2018)
Germination reduction (GR)	%	GR = GP of controls − GP of stress plants	Thabet et al. (2018)
Shoot length reduction (SLR)	mm	SLR = SL of controls − SL of stress plants	Partheeban et al. (2017)
Root length reduction (RLR)	mm	RLR = RL of controls − RL of stress plants	Thabet et al. (2018)
Germination stress tolerance index (GSTI)	%	$\mathrm{GSTI}=\frac{\mathrm{GP}\;\mathrm{under}\;\mathrm{salt}\;\mathrm{stress}\;\mathrm{conditions}\;}{\mathrm{GP}\;\mathrm{under}\;\mathrm{normal}\;\mathrm{conditions}}\times 100$	Partheeban et al. (2017)
Shoot length stress tolerance index (SLSTI)	%	$\mathrm{SLSTI}=\frac{\mathrm{SL}\;\mathrm{under}\;\mathrm{salt}\;\mathrm{stress}\;\mathrm{condtions}}{\mathrm{SL}\;\mathrm{under}\;\mathrm{normal}\;\mathrm{conditions}}\times 100$	Partheeban et al. (2017)
Root length stress tolerance index (RLSTI)	%	$\mathrm{RLSTI}=\frac{\mathrm{RL}\;\mathrm{under}\;\mathrm{salt}\;\mathrm{stress}\;\mathrm{condtions}}{\mathrm{RL}\;\mathrm{under}\;\mathrm{normal}\;\mathrm{conditions}}\times 100$	Partheeban et al. (2017)

## Data Availability

Not applicable.

## References

[1] Abdein M.A.E.-H. (2018). Genetic Diversity between Pumpkin Accessions Growing in the Northern Border Region in Saudi Arabia Based on Biochemical and Molecular Parameters. Egypt. J. Bot..

[2] Brown C.H., Luedeling E., Wichmann S., Epps P., Quinlan M., Lepofsky D. (2013). The paleobiolinguistics of domesticated squash (Cucurbita spp.). Explorations in Ethnobiology: The Legacy of Amadeo Rea.

[3] Ferriol M., Picó B., Nuez F. (2004). Morphological and Molecular Diversity of a Collection of *Cucurbita maxima* Landraces. J. Am. Soc. Hortic. Sci..

[4] Decker-Walters D.S., Walters T.W., Kiple K., Ornelas K.C. (2000). The Cambridge World History of Food.

[5] Le Floch E., Boulos L., Véla E. (2010). Catalogue Synonymique Commenté de la Flore Tunisie.

[6] Hamdi K., Palma D., Angelini P., Acciarri N., Tarchoun N., Sestili S. (2020). *Cucurbita maxima* Duch. population analysis: Relationship between Tunisian and Italian germplasm. J. Hortic. Sci. Biotechnol..

[7] Hamdi K., Ben-Amor J., Mokrani K., Mezghanni N., Tarchoun N. (2017). Assessment of the genetic diversity of some local squash (*Cucurbita maxima* Duchesne) populations revealed by agro-morphological and chemical traits. J. New Sci..

[8] Shahid M.A., Sarkhosh A., Khan N., Balal R.M., Ali S., Rossi L., Gómez C., Mattson N., Nasim W., Garcia-Sanchez F. (2020). Insights into the physiological and biochemical impacts of salt stress on plant growth and development. Agronomy.

[9] Sium A., Shawon A., Swapan K.R., Sun H.W., Kailas S.D., Abdullah S.M. (2019). Effect of salinity on the morphological, physiological and biochemical properties of lettuce (*Lactuca sativa* L.) in Bangladesh. Open Agric..

[10] Rouphael Y., Petropoulos S.A., Cardarelli M., Colla G. (2018). Salinity as eustressor for enhancing quality of vegetables. Sci. Hortic..

[11] Rogel J.A., Ariza F.A., Silla R.O. (2000). Soil salinity and moisture gradients and plant zonation in Mediterranean salt marshes of Southeast Spain. Wetlands.

[12] Rubio J.S., García-Sánchez F., Rubio F., Martínez V. (2009). Yield, blossom-end rot incidence, and fruit quality in pepper plants under moderate salinity are affected by K+ and Ca2+ fertilization. Sci. Hortic..

[13] Soltabayeva A., Ongaltay A., Omondi J.O., Srivastava S. (2021). Morphological, physiological and molecular markers for salt-stressed plants. Plants.

[14] Giordano M., Petropoulos S.A. (2021). Response and Defence Mechanisms of Vegetable Crops against Drought, Heat and Salinity Stress. Agriculture.

[15] Noctor G., Foyer C.H. (1998). Ascorbate and glutathione: Keeping Active Oxygen Under Control. Annu. Rev. Plant. Physiol. Plant. Mol. Biol..

[16] Saibi W., Feki K., Ben Mahmoud R., Brini F. (2015). Durum wheat dehydrin (DHN-5) confers salinity tolerance to transgenic *Arabidopsis* plants through the regulation of proline metabolism and ROS scavenging system. Planta.

[17] Sazzad Hossain M., Persicke M., Elsayed A.I., Kalinowski J., Dietz K.J. (2017). Metabolite profiling at the cellular and subcellular level reveals metabolites associated with salinity tolerance in sugar beet. J. Exp. Bot..

[18] Chaves M.M., Oliveira M.M. (2004). Mechanisms underlying plant resilience to water deficits: Prospects for water-saving agriculture. J. Exp. Bot..

[19] Kaur G., Asthir B. (2015). Proline: A key player in plant abiotic stress tolerance. Biol. Plant..

[20] Ashraf M., Harris P.J.C. (2013). Photosynthesis under stressful environments: An overview. Photosynthetica.

[21] Acosta-Motos J.R., Ortuño M.F., Bernal-Vicente A., Diaz-Vivancos P., Sanchez-Blanco M.J., Hernandez J.A. (2017). Plant responses to salt stress: Adaptive mechanisms. Agronomy.

[22] Mahlooji M., Seyed Sharifi R., Razmjoo J., Sabzalian M.R., Sedghi M. (2018). Effect of salt stress on photosynthesis and physiological parameters of three contrasting barley genotypes. Photosynthetica.

[23] Orlovsky N., Japakova U., Zhang H., Volis S. (2016). Effect of salinity on seed germination, growth and ion content in dimorphic seeds of *Salicornia europaea* L. (Chenopodiaceae). Plant. Divers..

[24] Petropoulos S.A., Daferera D., Polissiou M.G., Passam H.C. (2009). The effect of salinity on the growth, yield and essential oils of turnip-rooted and leaf parsley cultivated within the Mediterranean region. J. Sci. Food Agric..

[25] Golbashy M., Ebrahimi M., Khavari Khorasani S., Mostafavi K. (2012). Effects of drought stress on germination indices of corn hybrids (*Zea mays* L.). Electrnonic J. Plant. Breed..

[26] Almansouri M., Kinet J.M., Lutts S. (2001). Effect of salt and osmotic stresses on germination in durum wheat (*Triticum durum* Desf.). Plant. Soil.

[27] Flowers T.J., Munns R., Colmer T.D. (2015). Sodium chloride toxicity and the cellular basis of salt tolerance in halophytes. Ann. Bot..

[28] Khan N., Bano A., Babar M.A. (2019). The stimulatory effects of plant growth promoting rhizobacteria and plant growth regulators on wheat physiology grown in sandy soil. Arch. Microbiol..

[29] Savvas D., Lenz F. (2000). Effects of NaCl or nutrient-induced salinity on growth, yield, and composition of eggplants grown in rockwool. Sci. Hortic..

[30] Parvin K. (2015). Response of Tomato Plant Under Salt Stress: Role of Exogenous Calcium. J. Plant. Sci..

[31] Janghel D.K., Kumar K., Sunil R., Chhabra A.K. (2020). Genetic Diversity Analysis, Characterization and Evaluation of Elite Chickpea (*Cicer arietinum* L.) Genotypes. Int. J. Curr. Microbiol. Appl. Sci..

[32] Foti C., Khah E.M., Pavli O.I. (2019). Germination profiling of lentil genotypes subjected to salinity stress. Plant. Biol..

[33] Shin Y.K., Bhandari S.R., Jo J.S., Song J.W., Cho M.C., Yang E.Y., Lee J.G. (2020). Response to salt stress in lettuce: Changes in chlorophyll fluorescence parameters, phytochemical contents, and antioxidant activities. Agronomy.

[34] Othman Y., Al-Karaki G., Al-Tawaha A.R., Al-Horani A. (2006). Variation in Germination and Ion Uptake in Barley Genotypes under Salinity Conditions. World J. Agric. Sci..

[35] Mehr Z.S. (2013). Salt-induced changes in germination and vegetative stages of *Anethum graveolens* L. J. Stress Physiol. Biochem..

[36] Kandil A., Shareif A., Gad M. (2016). Effect of Salinity on Germination and Seeding Parameters of Forage Cowpea Seed. Res. J. Seed Sci..

[37] Viegas D.X., Piñol J., Viegas M.T., Ogaya R. (2001). Estimating live fine fuels moisture content using meteorologically-based indices. Int. J. Wildl. Fire.

[38] Yildirim E., Dursun A., Kumlay M.A., Güvenç Í. (2013). The effects of different salt, biostimulant and temperature levels on seed germination of some vegetable species. Acta Agrobot..

[39] Azeem A., Javed Q., Sun J., Nawaz M.I., Ullah I., Kama R., Du D. (2020). Functional traits of okra cultivars (Chinese green and Chinese red) under salt stress. Folia Hortic..

[40] Pavli O., Kempapidis K., Maggioros L., Foti C., Panagiotaki P., Khah E. (2021). Response of Lettuce Germplasm to Salt Stress at Different Developmental Stages. Ann. Agric. Crop Sci..

[41] Srivastava S., Sharma P.K. (2021). Effect of NaCl on chlorophyll fluorescence and thylakoid membrane proteins in leaves of salt sensitive and tolerant rice (*Oryza sativa* L) varieties. J. Stress Physiol. Biochem..

[42] Hasan B., Higano Y., Yabar H., Devkota M., Lamers J.P.A. (2015). Conservation Agriculture Practices in Salt-Affected, Irrigated Areas of Central Asia: Crop Price and Input Cost Variability Effect on Revenue Risks. Sustain. Agric. Res..

[43] Jamil M., Rha E.-S. (2004). The Effect of Salinity (NaCI) on the Germination and Seedling of Sugar Beet (*Beta vulgaris* L.) and Cabbage (*Brassica oleracea* L.). Plant. Resour..

[44] Pavli O.I. (2021). Effect of Salinity on Seed Germination and Seedling Development of Soybean Genotypes. Int. J. Environ. Sci. Nat. Resour..

[45] Abdoli M., Saeidi M., Azhand M., Jalali-honarmand S., Esfandiari E., Shekari F. (2013). The Effects of Different Levels of Salinity and Indole-3-Acetic Acid (IAA) on Early Growth and Germination of Wheat Seedling. J. Stress Physiol. Biochem..

[46] Ouji A., El-Bok S., Mouelhi M., Younes M., Kharrat M. (2015). Effect of salinity stress on germination of five Tunisian lentil *Lens culinaris* L. genotypes. Eur. Sci. J..

[47] Shin Y.K., Bhandari S.R., Cho M.C., Lee J.G. (2020). Evaluation of chlorophyll fluorescence parameters and proline content in tomato seedlings grown under different salt stress conditions. Hortic. Environ. Biotechnol..

[48] Abbas W., Ashraf M., Akram N.A. (2010). Alleviation of salt-induced adverse effects in eggplant (*Solanum melongena* L.) by glycinebetaine and sugarbeet extracts. Sci. Hortic..

[49] Kaya M.D., Ipek A., Öztürk A. (2003). Effects of different soil salinity levels on germination and seedling growth of safflower (*Carthamus tinctorius* L.). Turk. J. Agric. For..

[50] Benidire L., Daoui K., Fatemi Z.A., Achouak W., Bouarab L., Oufdou K. (2015). Effect of salt stress on germination and seedling of *Vicia faba* L. J. Mater. Environ. Sci..

[51] Alom R., Hasan M.A., Islam M.R., Wang Q.F. (2016). Germination characters and early seedling growth of wheat (*Triticum aestivum* L.) genotypes under salt stress conditions. J. Crop Sci. Biotechnol..

[52] Partheeban C., Chandrasekhar C.N., Jeyakumar P., Ravikesavan R., Gnanam R. (2017). Effect of PEG Induced Drought Stress on Seed Germination and Seedling Characters of Maize (*Zea mays* L.) Genotypes. Int. J. Curr. Microbiol. Appl. Sci..

[53] Hannachi S., Van Labeke M.C. (2018). Salt stress affects germination, seedling growth and physiological responses differentially in eggplant cultivars (*Solanum melongena* L.). Sci. Hortic..

[54] Du F., Shi H., Zhang X., Xu X. (2014). Responses of reactive oxygen scavenging enzymes, proline and malondialdehyde to water deficits among six secondary successional seral species in Loess Plateau. PLoS ONE.

[55] Ma J., Du G., Li X., Zhang C., Guo J. (2015). A major locus controlling malondialdehyde content under water stress is associated with *Fusarium* crown rot resistance in wheat. Mol. Genet. Genom..

[56] Zhou C., Wang Z.W., Han X.G., Yang Y.G., Busso C.A., Zhang Z., Yang Y.F. (2017). Effect of mixed salt stress on malondialdehyde, proteins and antioxidant enzymes of *Leymus chinensis* in three leaf colors. Phyton.

[57] Hnilickova H., Kraus K., Vachova P., Hnilicka F. (2021). Salinity stress affects photosynthesis, malondialdehyde formation, and proline content in *Portulaca oleracea* L. Plants.

[58] Xing J.-C., Dong J., Wang M.-W., Liu C., Zhao B.-Q., Wen Z.-G., Zhu X.-M., Ding H.-R., Zhao X.-H., Hong L.-Z. (2019). Effects of NaCl stress on growth of *Portulaca oleracea* and underlying mechanisms. Braz. J. Bot..

[59] Borsai O., Al Hassan M., Negrușier C., Raigón M.D., Boscaiu M., Sestraș R.E., Vicente O. (2020). Responses to salt stress in portulaca: Insight into its tolerance mechanisms. Plants.

[60] Jaarsma R., de Vries R.S.M., de Boer A.H. (2013). Effect of Salt Stress on Growth, Na+ Accumulation and Proline Metabolism in Potato (*Solanum tuberosum*) Cultivars. PLoS ONE.

[61] Sarabi B., Bolandnazar S., Ghaderi N., Ghashghaie J. (2017). Genotypic differences in physiological and biochemical responses to salinity stress in melon (*Cucumis melo* L.) plants: Prospects for selection of salt tolerant landraces. Plant. Physiol. Biochem..

[62] De la Torre-González A., Montesinos-pereira D., Blasco B., Ruiz J.M. (2018). Influence of the proline metabolism and glycine betaine on tolerance to salt stress in tomato (*Solanum lycopersicum* L.) commercial genotypes. J. Plant. Physiol..

[63] Zhani K., Mariem B.F., Fardaous M., Cherif H., Zhani K., Mariem B.F., Fardaous M., Cherif H. (2012). Impact of salt stress (NaCl) on growth, chlorophyll content and fluorescence of Tunisian cultivars of chili pepper (*Capsicum frutescens* L.). J. Stress Physiol. Biochem..

[64] Seroczyńska A., Antczak A., Kamińska K., Korytowska M., Korzeniewska A., Niemirowicz-szczytt K., Radomski A., Zawadzki J. (2014). Evaluation of the selected forms of winter squash (*Cucurbita maxima* Duch.) for the content of free sugars and polysaccharides. Polish J. Agron..

[65] Xu C., Mou B. (2015). Evaluation of lettuce genotypes for salinity tolerance. HortScience.

[66] Ekinci M., Yildirim E., Dursun A., Turan M. (2012). Mitigation of salt stress in lettuce (*Lactuca sativa* L. var. Crispa) by seed and foliar 24-epibrassinolide treatments. HortScience.

[67] ECPGR (2008). Minimum descriptors for *Cucurbita* spp., cucumber, melon and watermelon. Cucurbits Work. Gr..

[68] Monneveaux P., Nemmar M. (1986). Contribution à l’étude de la résistance à la sécheresse chez le blé tendre (*Triticum aestivum* L.) et chez le blé dur (*Triticum durum* Desf.): Étude de l’accumulation de la proline au cours du cycle de développement. Agronomie.

[69] Curtis O.F., Shetty K. (1996). Growth medium effects on vitrification, total phenolics, chlorophyll, and water content of in vitro propagated oregano clones. Acta Hortic..

